# Observation of an exceptional point in a two-dimensional ultrasonic cavity of concentric circular shells

**DOI:** 10.1038/srep38826

**Published:** 2016-12-13

**Authors:** Younghoon Shin, Hojeong Kwak, Songky Moon, Sang-Bum Lee, Juhee Yang, Kyungwon An

**Affiliations:** 1 School of Physics and Astronomy, Institute of Applied Physics, Seoul National University, Seoul 151-742, Korea; 2 Samsung Electronics Company, Hwaseong 445-330, Korea; 3 Korea Research Institute of Standards and Science, Daejeon 305-340, Korea; 4 Russia Science Seoul, Korea Electrotechnology Research Institute, Seoul 121-912, Korea

## Abstract

We report observation of an exceptional point in circular shell ultrasonic cavities in both theory and experiment. In our theoretical analysis we first observe two interacting mode groups, fluid- and solid-based modes, in the acoustic cavities and then show the existence of an EP of these mode groups exhibiting a branch-point topological structure of eigenfrequencies around the EP. We then confirm the mode patterns as well as eigenfrequency structure around the EP in experiments employing the schlieren method, thereby demonstrating utility of ultrasound cavities as experimental platform for investigating non-Hermitian physics.

A physical system can be described by a non-Hermitian Hamiltonian if the system is open or it has either absorptive loss or amplifying gain. One of the important properties of the non-Hermitian Hamiltonian is the existence of an exceptional point (EP), whose condition is satisfied when the coupling between interacting eigenstates is the same as their differential loss. At an EP, the eigenstates are degenerate in both eigenvalue and eigenfunction^1^,^2^,^3^. Consequently, it exhibits unusual properties such as branch-point topology, eigenstate exchange when encircled parametrically and breakdown of adiabaticity when encircled dynamically^4^,^5^,^6^.

EP’s have been observed in various physical systems such as microwave billiards^7^,^8^, deformed microcavities^9^, acoustic waves propagating in media of anisotropic thermoelasticity^10^, an atom-cavity quantum composite^11^, coupled-disk lasers^12^ and exciton-polariton billiards^13^. In particular, it is known that EP’s in optical systems show many interesting features such as divergent Petermann factor^14^,^15^, reversal of the pump dependence in lasing^16^ and enhanced detection sensitivity^17^.

Even though the optical microcavities have been widely used in studying EP’s as well as other non-Hermitian properties, they have some weak points. For example, spatial mode patterns in an optical microcavity would show many interesting features related to quantum chaos and intermode interactions^18^,^19^,^20^,^21^. However, it is almost impossible to visualize the mode patterns experimentally in optical microcavities without introducing scatterers, which inevitably disturb the system. For this reason, the mode characteristics have been studied mostly in terms of the far-field patterns and emission spectra.

To supplement this limitation, we propose to exploit an ultrasonic cavity, in which the ultrasonic field can be easily measured by using the schlieren method^22^,^23^. This technique has been widely used in visualizing fluid motion around objects such as bullet bow shockwave and thermal flume from a thermal source. Likewise, with the schlieren method we can visualize the refractive index modulation caused by ultrasonic waves in a transparent medium.

Previously, Chinnery and Humphrey studied the resonance properties of a stadium-shaped ultrasonic cavity by using the schlieren method, presenting various modes patterns and their statistical properties^24^. They also reported mode overlapping in a fluid-filled cavity^25^ as well as shape-dependence of modes in elliptical cavities^26^. Quite recently, multiple EP’s in air-filled four coupled acoustic cavities have been investigated with wall-mounted microphones^27^ without observing mode patterns. However, both mode patterns and resonance spectrum around an EP have not been studied in acoustic cavities so far.

In this paper, we investigate resonance properties – mode patterns and resonance spectrum – of concentric ultrasonic shell cavities in both theory and experiment. By carrying out theoretical calculations, we show that there exist two interacting mode groups, fluid- and solid-based modes. We then explicitly show the existence of an EP exhibiting a complex-square-root-like topological structure of eigenfrequencies around it. Moreover, we present the experimental results obtained with the schlieren method and confirm our theoretical predictions, thereby demonstrating the utility of ultrasonic cavities for studying the physics of non-Hermitian systems.

Let us first consider a 2D ultrasonic cavity with concentric circular shells as depicted in Fig. 1. The shell cavity has three sub-regions: inner fluid, a solid shell, and outer fluid. This cavity is one of the simplest coupled ultrasonic cavities which allow ease in both theoretical analysis and experimental realization. Because of the rotational symmetry, resonant modes of the cavity can be easily found analytically. In the frequency domain, the harmonic ultrasound fields are described by the Helmholtz equation in the fluid and by Cauchy-Navier equation in the solid. Resonant normal modes of the shell cavity are then given by nontrivial solutions of a matrix equation det[**M**(*ω*_res_)] = 0 derived from the wave equations as explained in detail in Methods. The complex frequencies *ω*_res_ = *ω*_*r*_ + *iω*_*i*_ (*ω*_*r*_ > 0, *ω*_*i*_ < 0) satisfying det [**M**(*ω*_res_)] = 0 are the resonant frequencies of the normal modes.

## Results

**Figure 1:**
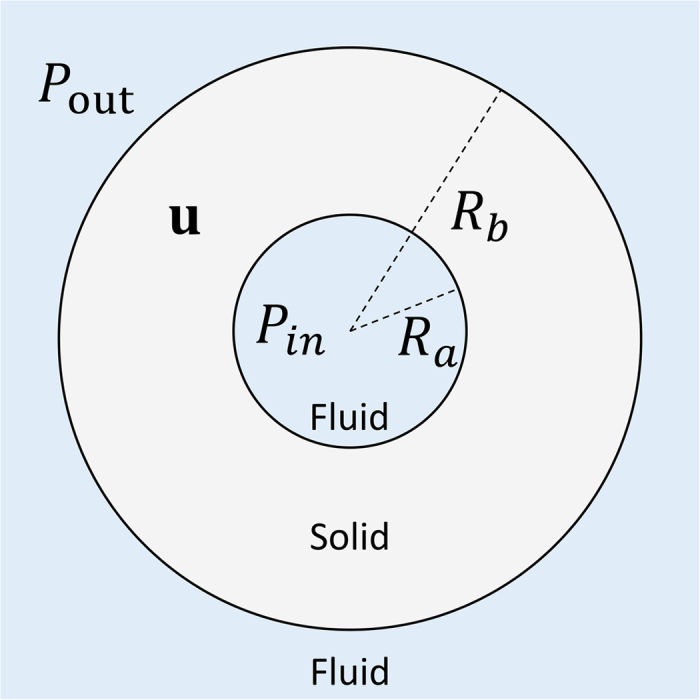
Structure of our 2D shell cavity. It consists of three sub-regions: inner fluid, a solid shell, and outer fluid. Ultrasound fields are described in terms of pressure *P*_in_ and *P*_out_ inside the inner and outer fluid and displacement **u** inside the solid.

We have solved the matrix equation near *k*_*f*_*R*_*a*_ = 20 and obtained several resonant frequencies as well as the wavefunctions of the modes. Here *k*_*f*_ is the wavenumber of the sound wave in the fluid and *R*_*a*_ is the inner radius of the shell as defined in Fig. 1. We selected aluminum as the solid material and water as the fluid. The characteristic constants used in the calculation are listed in Table 1. In this calculation we find that two groups of modes exist in the shell cavity. One group, called fluid-based mode (FBM), is mostly localized in the internal fluid region and the other group, called solid-based mode (SBM), is mostly localized within the solid shell.

**Figure 2:**
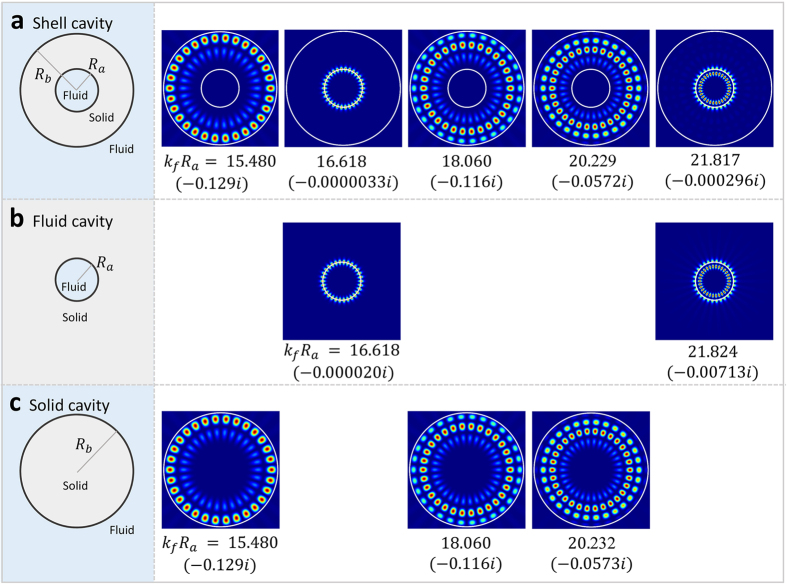
Decomposition of the shell-cavity modes into FBM and SBM. The white circles indicate the shell boundaries. Distributions |*P*|^2^ inside the fluid and |*σ*_*rr*_|^2^ inside the shell are plotted for modes with *R*_*b*_ = 3*R*_*a*_ and *m* = 15. The values in the parentheses are the imaginary parts of the size parameters *k*_*f*_*R*_*a*_.

An example of decomposing the modes into FBM and SBM is shown in Fig. 2. The shell cavity modes are presented in the first row of Fig. 2, where we plotted the pressure field intensity |*P*|^2^ inside the fluid and the stress tensor |*σ*_*rr*_|^2^ in the radial direction in the solid shell. It is found that modes with Re[*k*_*f*_*R*_*a*_] = 15.480, 18.060, 20.229 are localized within the shell, while the other modes are localized inside the internal fluid. As a consequence, we can consider the FBM’s as the modes of a separate cavity whose external fluid is replaced by infinite solid. In a similar way, SBM’s can be considered as the modes of another cavity whose internal fluid is replaced by solid. The adequacy of the above mode decomposition is evidently seen in Fig. 2(b) and (c).

We investigated the mode interactions based on the decomposition of shell modes into FBM’s and SBM’s. If we vary the outer radius *R*_*b*_ with the inner radius *R*_*a*_ fixed, the resonance frequencies of FBM’s are almost invariant. It is because the modes localized in the internal fluid are hardly affected by the changes of the outer shell boundary. On the other hand, the frequencies of SBM’s are inversely proportional to the outer radius *R*_*b*_ of the shell because the size parameter (*k*_*f*_*R*_*b*_ for SBM) is a constant for a mode regardless of the system size. Accordingly, FBM’s and SBM’s can move closer to or move away from each other with varying *R*_*b*_, allowing interactions between two groups of modes across the inner boundary.

**Figure 3:**
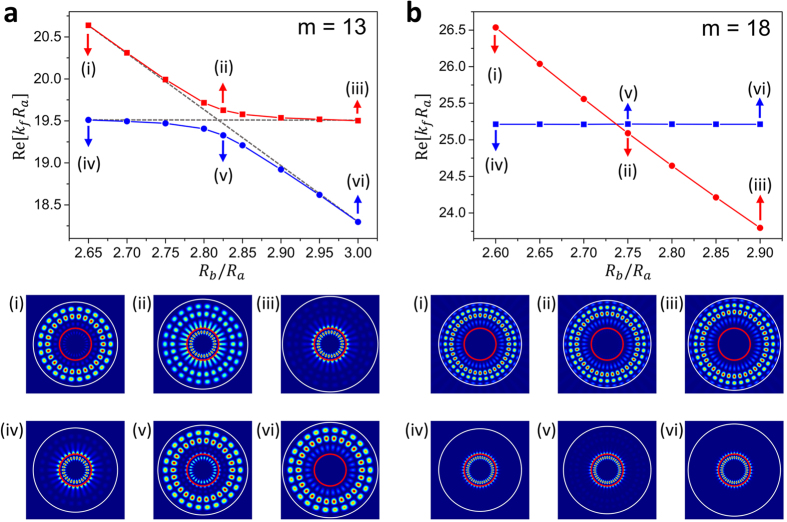
Avoided crossing and mode crossing. Dotted lines are diabatic lines. The distribution |*P*|^2^ inside the fluid, and |*σ*_*rr*_|^2^ inside the shell are plotted as in Fig. 2. The outer boundary of the shell is colored in white whereas the inner boundary is colored in red in order to facilitate direct comparison with the experimental results in Fig. 7. (**a**) For *m* = 13, one can observe mode pattern exchange and mode mixing by following the path of instantaneous solutions. (**b**) For *m* = 18, however, FBM and SBM cross each other and no mode exchange and mode mixing observed.

This behavior is shown in Fig. 3. As mentioned above, FBM’s are not affected by the change of *R*_*b*_/*R*_*a*_ with *R*_*a*_ fixed. When the two mode groups are far apart, Re[*k*_*f*_*R*_*a*_] values of the FBM’s more or less follow a constant horizontal line, which is often called the diabatic transition line. Similarly, Re[*k*_*f*_*R*_*a*_] values of SBM’s follow another diabatic transition line with the inverse dependence on *R*_*b*_. As the ratio *R*_*b*_/*R*_*a*_ is varied for different angular quantum numbers *m*’s, FBM’s and SBM’s repel (avoided crossing, AC) or cross (mode crossing, MC) each other near the crossing point of the diabatic lines. Here, the angular quantum number *m* equals a half of the number of anti-nodes of the wavefunction in the direction of the azimuthal angle. It is also proportional to the angular momentum 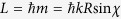 of a fictitious particle associated with the wave solution in the semi-classical limit with *χ* the incident angle of the particle on the circular boundary of radius *R*. Note that the angular quantum number *m* was used as an internal system parameter in the previous studies^9^,^28^. In the case of MC, FBM’s and SBM’s just follow their diabatic lines. In the case of AC, however, FBM’s and SBM’s do not follow their diabatic lines but follow the paths of instantaneous solutions accompanying a mode gap which is approximately proportional to the strength of the interaction between two groups. By following these paths, spatial mode patterns change from FBM to SBM or vice versa. Such mode pattern exchange has been experimentally observed in other systems such as in microwave billiards^7^ and in exciton-polariton billiards^13^.

### Exceptional point

Exceptional point (EP) is a singular point in parametric space where two interacting modes coalesce into one mode. EP condition is satisfied when the coupling equals their differential loss. Occurrence of an EP can be easily understood in a simple 2 × 2 matrix model. Let us consider a non-Hermitian Hamiltonian given by


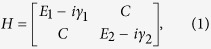


where unperturbed modes have real energy *E*_1_, *E*_2_ and decay rates *γ*_1_, *γ*_2_ (*γ*_1_ > *γ*_2_). The coupling *C* between the modes is assumed to be real. After diagonalization of the Hamiltonian, we get eigenvalues 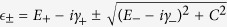, where *E*_±_ = (*E*_1_ ± *E*_2_)/2 and *γ*_±_ = (*γ*_1_ ± *γ*_2_)/2. When *E*_1_ = *E*_2_ (i.e., *E*_−_ = 0), 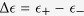 depends on the coupling *C* and the differential decay rate *γ*_−_. If *γ*_−_ > *C*, then the energy difference is given by 
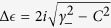
. Therefore, when we vary the detuning *E*_−_ across zero, the real parts of the energy cross but the imaginary parts repel each other. If *γ*_−_ < *C*, on the other hand, we get 
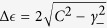
, which means avoided crossing in real parts and crossing in imaginary parts as the detuning *E*_−_ is varied. Lastly, if *γ*_−_ = *C*, the real and imaginary parts of two modes have the same values. Moreover, two eigenfunctions become the same in this case, differently from the usual energy degeneracy. This coalesced mode is called an EP mode.

**Figure 4:**
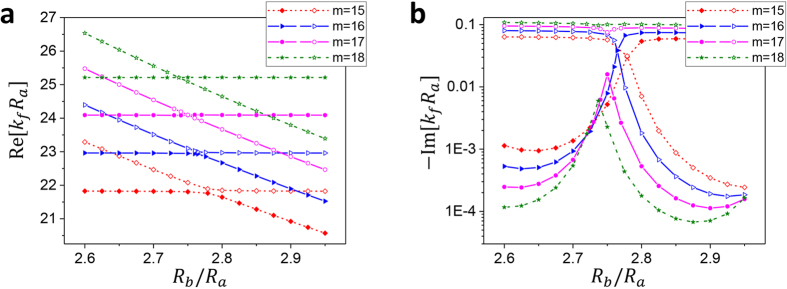
Transition between MC and AC. (**a**) Real parts Re[*k*_*f*_*R*_*a*_] and (**b**) imaginary parts Im[*k*_*f*_*R*_*a*_] of the eigenvalues and for *m* = 15, …, 18. Solid (open) symbols represent the modes followed from FBM’s (SBM’s) in the lower (*R*_*b*_/*R*_*a*_) range. FBM’s and SBM’s with *m* = 17, 18 cross each other in real parts but repel in imaginary parts. For smaller *m*’s, FBM’s and SBM’s repel each other in real parts showing AC, but the imaginary parts cross each other.

**Figure 5:**
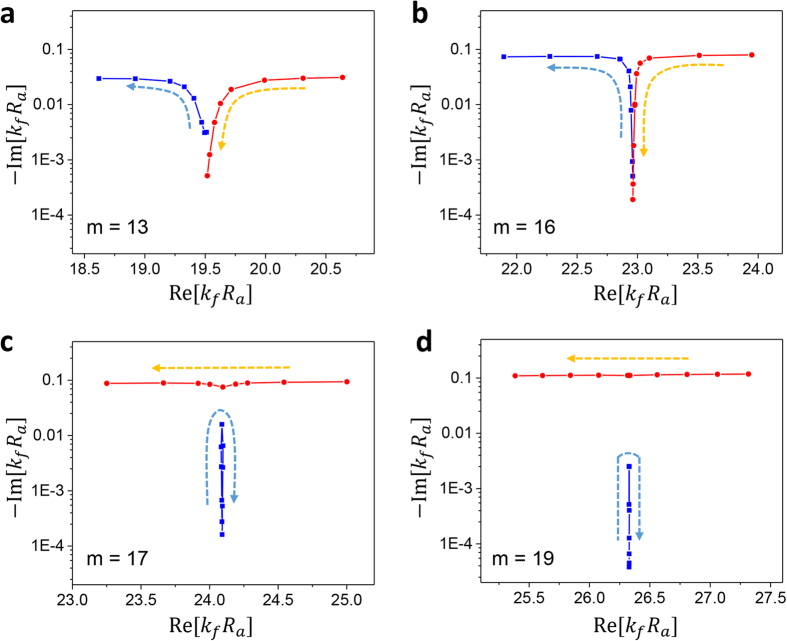
Trajectories of the complex eigenvalues as *R*_*b*_/*R*_*a*_ is varied. (**a**) *m* = 13, (**b**) *m* = 16, (**c**) *m* = 17 and (**d**) *m* = 19. The arrows indicate the direction of the trajectories as *R*_*b*_/*R*_*a*_ is increased from 2.60 to 2.95.

In Fig. 4(a) and (b), the resonance modes of the shell cavity are plotted as *R*_*b*_/*R*_*a*_ is varied. Solid (open) symbols represent the modes followed from FBM’s (SBM’s) in the lower (*R*_*b*_/*R*_*a*_) range. In Fig. 4(a), real eigenvalues Re[*k*_*f*_*R*_*a*_] are plotted whereas in Fig. 4(b) the imaginary parts are plotted. In these plots, we observe a transition between MC and AC. When *m* = 17, 18, FBM’s and SBM’s are undergoing MC (AC) in real (imaginary) parts. For the smaller *m* values, the modes are undergoing AC (MC) in real (imaginary) parts. In Fig. 5(a–d), the trajectories of the complex eigenvalues are plotted as *R*_*b*_/*R*_*a*_ value is increased. Blue (red) dots are followed from the FBM’s (SBM’s) in the lower *R*_*b*_/*R*_*a*_ range.

**Figure 6:**
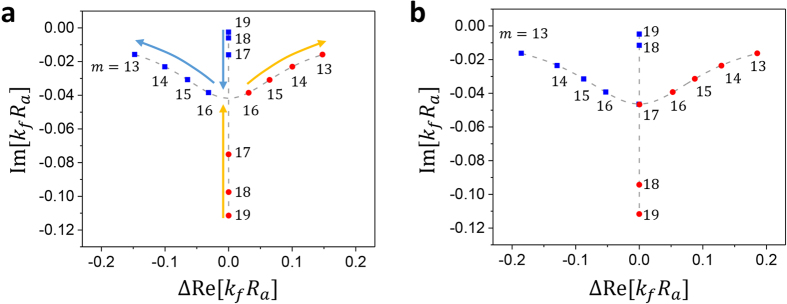
Complex eigenvalues at the crossing points of the diabatic lines of Re[*k*_*f*_*R*_*a*_] for various *m*’s. Horizontal axes represent the relative Re[*k*_*f*_*R*_*a*_] values of the interacting modes. (**a**) Results for the parameters in Table 1. (**b**) Results for a slightly different sound velocity *v*_*l*_ = 1518.03 m/s in fluid. Arrows in (**a**) indicate the moving directions of the eigenvalues as we increase *v*_*l*_, indicating *m* = 17 modes become the EP mode in (**b**).

It is evident that an EP exists somewhere between *m* = 16 and 17 when 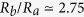 in the parameter space. Note the internal parameter *m* controlling *E*_−_(detuning) is an integer and thus discrete. For this reason it is difficult to hit the exact position of an EP in the (*m, R*_*b*_/*R*_*a*_) parameter space. However, it is in principle possible to reach the EP by changing a continuous system parameter such as density of fluid, instead of *m*, which is accessible by mixing two different types of fluids. For example, in Fig. 6, complex eigenvalues at crossing points of the diabatic lines for Re[*k*_*f*_*R*_*a*_] are displayed. Figure 6(a) is the results for the parameters in Table 1. As we mention above, it is impossible to reach an EP with only varying the discrete parameter *m*. If we slightly change the sound velocity in the fluid – by changing the Lamé’s parameters – as in Fig. 6(b), however, we can hit the EP accurately. In this case, *m* = 17 modes become an EP mode. Another way to reach an EP is to include additional loss in the fluid, which can be simulated by introducing a complex sound velocity.

### Experiment

**Figure 7:**
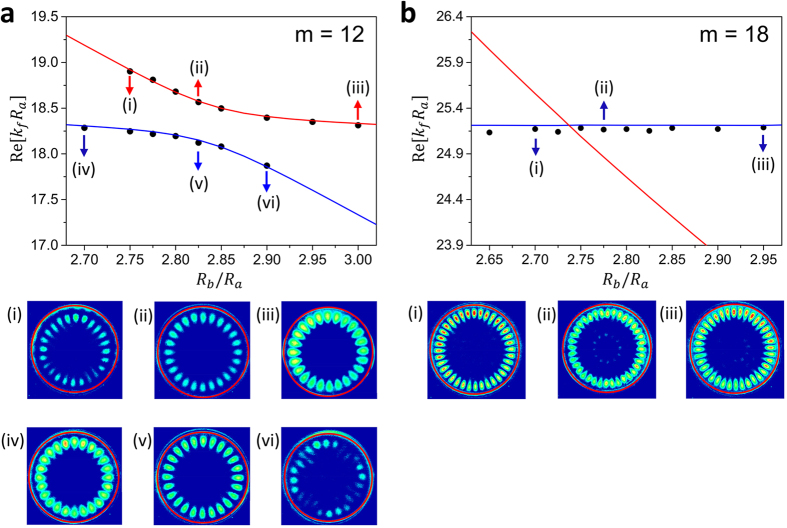
Experimentally observed resonances and their mode patterns. Black dots are the experimental data. Blue and red lines represent the theoretical expectations. In the schlieren images, red circles indicate the inner boundary of the shell. (**a**) Avoided crossing between FBM and SBM. Due to the limitation of the schlieren method, mode pattern in the solid shell could not be visualized. However, one can still observe mode pattern exchange by noticing the reduction of mode intensity following path (iii) → (ii) → (i) or (iv) → (v) → (vi). (**b**) Mode crossing result. Unperturbed SBM’s could not be visualized because there is little mode mixing between FBM’s and SBM’s. Measurement error bars are smaller than the dot size in (**a**) and (**b**).

We now present our experimental results to verify our theoretical predictions. Frequencies and mode patterns of resonance modes obtained with the schlieren method are shown in Fig. 7, where experimental data are marked by black dots. Blue and red lines are the theoretical paths of instantaneous solutions, followed from FBM and SBM in the lower *R*_*b*_/*R*_*a*_ region, respectively. We observe a good agreement between theory and experiment. Mode patterns visualized by the schlieren method are displayed below the mode spectrum. As already shown in the theoretical analysis or in Fig. 3(a), we observe AC in the spectrum as well as the mode pattern exchange in Fig. 7(a). Note that the intensity of the mode pattern in the fluid is gradually reduced if we follow the path (iii) → (ii) → (i) or (iv) → (v) → (vi). This is because unperturbed SBM’s do not have any spatial distributions in the fluid. In Fig. 7(b), however, we observe FBM’s with a constant Re[*k*_*f*_*R*_*a*_]. In this MC case, there is neither mode splitting nor noticeable spatial mode pattern mixing. As a result, mode patterns of the SBM’s could not be visualized because they have negligible spatial distribution in the inner fluid. In addition, the mode patterns of the FBM’s are hardly affected by the change of *R*_*b*_/*R*_*a*_ as expected in the theoretical analysis or in Fig. 3(b).

**Figure 8:**
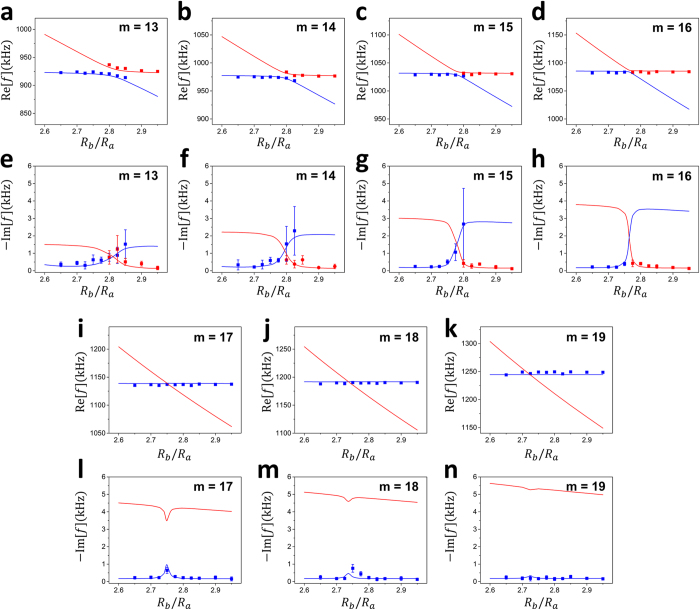
Experimental observation of an EP. Blue (red) lines are theoretically expected resonances followed from FBM’s (SBM’s) in the lower *R*_*b*_/*R*_*a*_ range. Solid dots are experimental observations. (**a**–**d**) and (**i**–**k**): Real parts of the resonance frequencies. Error bars are smaller than the size of the symbols. (**e**–**h**) and (**l**–**n**): Imaginary parts of the resonance frequencies extracted from the linewidths of the observed spectra. An EP exists between *m* = 16 and *m* = 17 with 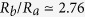. As shown in the theoretical analysis, one can reach an EP by adjusting the sound velocity or the medium loss continuously.

In Fig. 8, the experimentally observed resonances (symbols) supporting the existence of an EP are shown with the theoretical expectations (lines). For theoretical calculation, we used a complex sound velocity *v*_*f*_ = (1485 − *i*0.22)m/s in the fluid in order to account for the scattering and absorption loss present in the experiment. This small imaginary component corresponds to a medium-loss quality factor 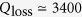, consistent with the loss-broadened linewidths of otherwise high-Q modes in the experiment. It is seen that AC (MC) occurs for *m* ≤ 16 while MC (AC) occurs for *m* ≥ 17 in the real (imaginary) parts of resonance frequencies. Although we can measure only modes with spatial distribution in the fluid by the schlieren method, the transition from AC to MC can be clearly seen in Fig. 8 as *m* is increased. This observation implies the existence of an EP with 16 < *m* < 17 and 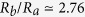.

## Discussion

In both theory and experiment, we have observed the transition from AC to MC by increasing angular quantum number *m*. This transition is due to the reduced *C* compared to *γ*_−_. The transition can be analyzed in more details as follows.

If *m* is increased with the radial quantum number *l* fixed, the size parameter Re[*k*_*f*_*R*_*a*_] of both FBM and SBM increases since the size parameter is approximately equal to the number of wavelengths fitting the inner circumference of the shell. Moreover, the distributions of FBM and SBM are shifted to the internal and external boundaries, respectively, corresponding to an increased incident angle of waves on the boundaries (recall *m* = *kR*sin*χ*). As a result, the loss of FBM is reduced whereas that of SBM is increased. The coupling decreases much more than the loss of FBM. The reason is as follows. As *m* is increased, the distribution of FBM in the solid region is reduced because of the decreased loss of FBM, and at the same time the distribution of SBM further shifts to the external boundary. Therefore, the wavefunction overlap between FBM and SBM is greatly reduced, resulting in the coupling much more decreased than the loss of FBM. Therefore, we can induce a transition from AC to MC by increasing the angular quantum number *m*.

It is apparent that the schlieren method cannot visualize the mode patterns inside the opaque shell (aluminium). As shown for *m* ≥ 17 in Fig. 8, this limitation is pronounced in the weak-coupling regime. However, with smaller *m* values, for which the coupling is strong, it was possible to measure the SBM-like modes partially even quite away from the *R*_*b*_/*R*_*a*_ point where the diabatic lines cross. It is because the SMB-like modes still have some distribution in the internal fluid due to the mode mixing arising from the intermode interaction between SBM and FBM. As a result, we could observe AC in real eigenvalues despite the limitation of the schlieren method. Note it is still impossible to visualize *unperturbed* SBM’s in the opaque solid with the schlieren method since little mode-mixing exists with FBM’s. This limitation, however, can be easily overcome by adopting transparent solid such as glass or acrylic. Ultrasound cavities made of fluid enclosed in transparent solid would thus be a promising platform for studying intermode interactions in non-Hermitian systems. In particular, there are many interesting phenomena expected to occur near EP’s such as adiabaticity breaking when an EP is dynamically encircled^4^,^5^,^6^,^29^,^30^, chirality of EP modes^31^ and mode evolution near a triple EP^32^. We expect these phenomena can be effectively investigated without disturbing the system by using our approach in terms of both eigenvalues and eigenfunctions.

## Methods

### Solving wave equations numerically

The shell cavity has three sub-regions: inner fluid, a solid shell, and outer fluid (see Fig. 1). In the frequency domain, the harmonic ultrasound fields are described by the Helmholtz equation in the fluid and by Cauchy-Navier equation in the solid:


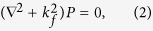



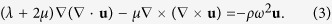


Here *P* is the pressure field in the fluid and **u** is the displacement vector in the solid. The wavenumber *k*_*f*_ of sound wave in the fluid is defined as *k*_*f*_ = *ω*/*v*_*f*_ where *ω* and *v*_*f*_ are the angular frequency and the sound velocity in the fluid. In addition, *λ* and *μ* are the Lamé’s first and second parameters of the solid, respectively, and *ρ* is the density of the solid.

Inside the fluid surrounded by the solid shell, two-dimensional solution for *P* is given by a simple form 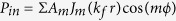, where *J*_*m*_ is the Bessel function of order *m*. In the solid shell, it is conventional to introduce scalar and vector potential *φ* and 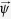, from which **u** is given by 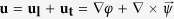. Obviously 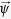 has only *z* component in a 2D system described in *x* and *y* coordinates. By substituting the potential form of **u** in Eq. (3) and after rearranging terms according to their polarization, we obtain two Helmholtz equations for *φ* and **ψ** as


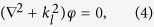



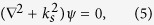


where *k*_*l*_ and *k*_*s*_ are the longitudinal and shear wavenumbers which are defined as 
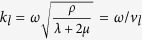
 and 
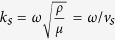
 with *v*_*l*_ the longitudinal and *v*_*s*_ the shear velocity. Therefore, the solutions for the Eqs (4) and (5) are of the form


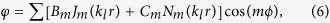



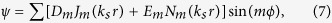


where *N*_*m*_ is the Neumann function of order *m*. Outside the shell, the pressure field is also found from Eq. (2), but in order to satisfy the outgoing wave condition we take the first kind Hankel function instead of the Bessel function: 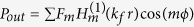.

Our goal now is to find the resonant frequencies of the normal modes. To do this, we need six boundary conditions for the six unknowns {*A*_*m*_, *B*_*m*_, …, *F*_*m*_} for a given *m*. The boundary conditions are as follows. The first is the continuity of normal components of the stress, which is just the equilibrium of surface normal forces to maintain the interface. Next is the continuity of the displacement, *i.e.*, the solid and the fluid should contact each other all the time. The last is that the tangential stress at the inner (*r* = *R*_*a*_) and outer (*r* = *R*_*b*_) interfaces should vanish because there cannot be shear stress in the fluid. These conditions are explicitly given by (1) *σ*_*rr*_(*R*_*a*_) = −*P*_*in*_(*R*_*a*_), *σ*_*rr*_(*R*_*b*_) = −*P*_*out*_(*R*_*b*_), (2) 
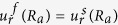
, 
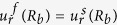
, (3) *σ*_*rϕ*_(*R*_*a*_) = *σ*_*rϕ*_(*R*_*b*_) = 0, respectively, where *σ*_*ij*_ is the stress tensor within the shell defined as


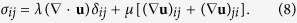


The superscripts *f* and *s* in the displacement *u* refer to fluid and solid. Indices *i, j* in the stress tensor *σ* denote orthogonal coordinates *r* and *ϕ*.

After substituting the expressions for **u** and *P* into the boundary conditions, one finds six linear equations for six unknowns which depend on the complex frequency *ω*. Accordingly, those equations can be written in the 6 × 6 matrix form **M**(*ω*)**b** = **0** for a given *m*, where **b** consists of the field coefficients {*A*_*m*_, …, *F*_*m*_}.

In the cylindrical coordinates, the surface-normal displacements and the components of the stress tensor are easily found to be as follows.


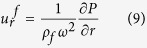



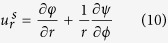



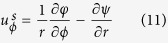



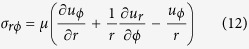



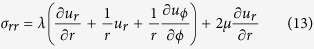


By substituting *P, φ, ψ* for the boundary conditions given in the main text and after some algebra, we get six homogeneous linear equations for the coefficients {*A*_*m*_, …, *F*_*m*_} of the field.


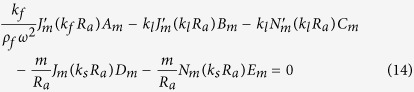



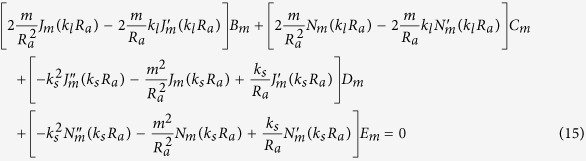



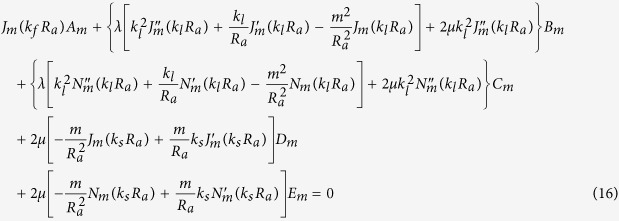



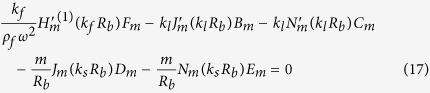



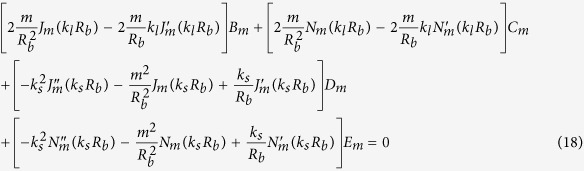



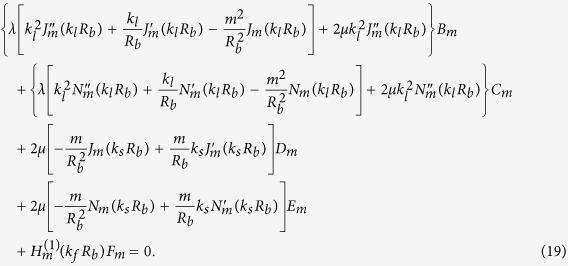


The equations are summarized to a simple matrix form **M**(*ω*)**b** = **0**, where **b** is a column vector consisting of the field coefficients {*A*_*m*_, …, *F*_*m*_}. As mentioned in the main text, to find nontrivial solutions one need to search complex *ω*’s such that **det**(**M(w)**) = 0. These *ω*’s can be found by using the Newton-Raphson method in complex space, as in an optical microcavity^33^. Because we take the convention that the fields have the form of *e*^*i*(**k**·**r**−**wt**)^, *ω* is obviously expressed by *ω* = *ω*_*r*_ + *iω*_*i*_ = *ω*_*r*_ − *i*|*ω*_*i*_| (*ω*_*i*_ is negative), where *ω*_*r*_ mainly determines the spatial distribution of the field and (−*ω*_*i*_) gives the decay rate of the resonant mode. Then the quality factor *Q* of a mode is given by *Q* = −*ω*_*r*_/2*ω*_*i*_.

### Experimental setup

We fabricated aluminium shells with *R*_*a*_ = 5 mm and *R*_*b*_ ranging from 2.65*R*_*a*_ to 3.0*R*_*a*_ in total of 11 steps. The surface roughness is about 10*μ*m, which is negligible compared to the sound wavelength of interest (order of 1 mm). The cavity is immersed in distilled water. The water is first heated to the boiling temperature to remove dissolved air. It is then rapidly cooled down to the room temperature by a immersion chiller in order to avoid re-dissolving of air. In addition, we cover the surface of water with polyethylene spheres for the same reason. With this procedure, small air bubbles which act as scatterers of the sound waves are mostly eliminated, allowing high-*Q* modes with *Q* ~ 10^4^.

**Figure 9:**
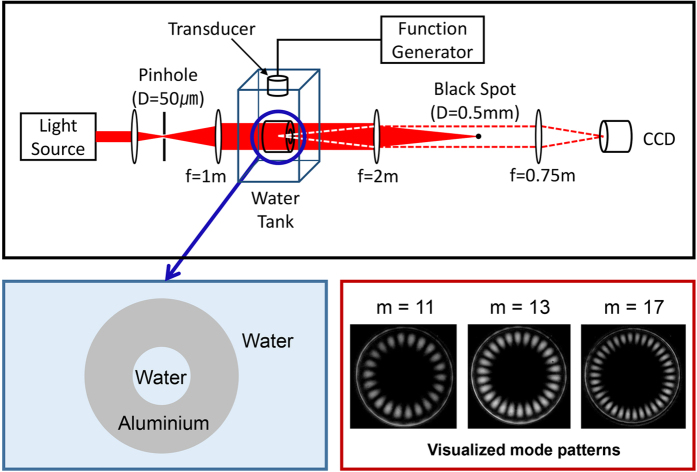
Schematic of the experimental setup. The schlieren method is used to visualize resonant modes patterns. Observed mode patterns of some modes with radial quantum number 2 are shown as examples. Bright regions in the mode patterns represent the anti-nodes of the eigenfunctions. Bright circles are the inner boundaries of the shell cavity.

The cavity modes are excited by an immersion ultrasonic transducer which is driven by a function generator with an RF amplifier (Fig. 9). The driving sine wave frequency is scanned in the range of 800 kHz–1.3 MHz. Spatial intensity patterns are measured by using the schlieren method, which is widely used to visualize the refractive index modulations in transparent media. It is well established that the schlieren image represents the sonic pressure intensity |*P*|^2^ at low pressure^34^. When the driving frequency is on resonance with a FBM, one can observe a bright image of the pressure field in the internal fluid. In addition, the spectrum of FBM’s can be obtained by integrating the pressure field distribution seen in the schlieren image as a function of the excitation frequency. Therefore, with our setup, we are able to measure the mode patterns as well as the mode spectrum simultaneously. Spatial mode patterns around an EP have been observed in microwave billiards before by scanning a perturbative probe^35^. Our setup does not need such a physical probe, which is known to introduce unwanted perturbation to the system^36^.

### Inclusion of medium loss

In actual experiments, scattering and absorption loss inevitably occurs in media, mostly in the fluid in our experiment. The loss in the fluid can be included in our theoretical calculation by introducing an imaginary component *v*_*i*_(<0) in the longitudinal sound velocity *v*_*f*_ in the fluid. Note *k*_*f*_ in Eqs (14), (16), (17) and (19) are replaced with


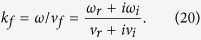


The new matrix equation **M**(*ω*)**b** = **0** is solved for complex frequency *ω*, which is now given by


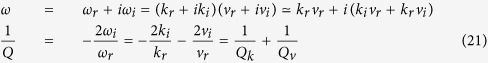


where *k*_*i*_, *v*_*i*_ < 0 is assumed. This equation indicates that the total loss 1/*Q* is composed of 1/*Q*_*k*_ = −2*k*_*i*_/*k*_*r*_ accounting for the wave-tunneling loss and 1/*Q*_*v*_ = −2*v*_*i*_/*v*_*r*_ for absorption and scattering loss in the medium. The quality factor *Q*_*v*_ corresponding to the medium loss has been estimated to be approximately 3400 from the observed linewidth of otherwise high-Q mode (*Q*_*k*_ ~ 10^5^) in our experiment. The estimated medium loss is found to be consistent with our choice of *v*_*i*_ = −0.22 m/s.

## Additional Information

**How to cite this article**: Shin, Y. *et al*. Observation of an exceptional point in a two-dimensional ultrasonic cavity of concentric circular shells. *Sci. Rep.* **6**, 38826; doi: 10.1038/srep38826 (2016).

**Publisher's note:** Springer Nature remains neutral with regard to jurisdictional claims in published maps and institutional affiliations.

## Figures and Tables

**Table 1:** Characteristic constants of the materials used in our calculation.

	*λ* (N/m^2^)	*μ* (N/m^2^)	*ρ* (kg/m^3^)	*vl* (m/s)	*vs* (m/s)
water	2.201 × 10^9^	0	998	1485	NA
aluminium	5.494 × 10^10^	2.645 × 10^10^	2700	6320	3130

Here *λ* and *μ* are the Lamé’s first and second parameters of the solid, respectively, and they are derived from the longitudinal velocity *v*_*l*_ and shear velocity *v*_*s*_ in the solid by using the relation 
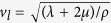
 and 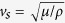 with *ρ* the density of the solid. Parameter values of *ρ, v*_*l*_ and *v*_*s*_ are from ref. 37.
